# Postural changes in blood pressure among patients with diabetes attending a referral hospital in southwestern Uganda: a cross-sectional study

**DOI:** 10.1186/s12872-021-02022-5

**Published:** 2021-04-27

**Authors:** Richard Migisha, David Collins Agaba, Godfrey Katamba, Jennifer Manne-Goehler, Anthony Muyingo, Mark J. Siedner

**Affiliations:** 1 Department of Physiology, Mbarara University of Science and Technology, P.O. Box 1410, Mbarara, Uganda; 2 Department of Physiology, King Ceasor University, Kampala, Uganda; 3 Divsion of Infectious Diseases, Brigham and Women’s Hospital, Harvard Medical School, Boston, USA; 4 Department of Internal Medicine, Mbarara University of Science and Technology, Mbarara, Uganda; 5 Department of Medicine, Massachusetts General Hospital, Boston, USA

**Keywords:** Orthostatic hypotension, Orthostatic hypertension, Diabetes, Uganda

## Abstract

**Background:**

Orthostatic hypotension (OH) and orthostatic hypertension (OHT) are often unrecognized in clinical care for diabetic individuals, yet they are associated with increased risk for adverse cardiovascular outcomes. We aimed to determine the prevalence of the abnormal orthostatic blood pressure (BP) responses, and associated factors among diabetic individuals in ambulatory care for diabetes in southwestern Uganda.

**Methods:**

We conducted a cross-sectional study among diabetic individuals aged 18–65 years at Mbarara Regional Referral Hospital, southwestern Uganda from November 2018 to April 2019. We obtained demographic and clinical data including a detailed medical history, and glycemic profile. BP measurements were taken in supine position and within 3 min of standing. We defined OH in participants with either ≥ 20 mmHg drop in systolic BP (SBP) or ≥ 10 mmHg drop in diastolic BP (DBP) after assuming an upright position. OHT was defined in participants with either a ≥ 20 mmHg rise in SBP, or ≥ 10 mmHg rise in DBP after assuming an upright position. Multivariate logistic regression was used to identify factors associated with OH and OHT.

**Results:**

We enrolled 299 participants, with a mean age of 50 years (SD ± 9.8), and mean HbA1c of 9.7% (SD ± 2.6); 70% were female. Of the 299 participants, 52 (17.4%; 95% CI 13.3–22.2%) met the definition of OH and 43 (14.4%; 95% CI 10.6–18.9%) were classified as having OHT. In multivariable models, factors associated with diabetic OH were older age (OR = 2.40 for 51–65 years vs 18–50 years, 95% CI 1.02–5.67, *P* = 0.046), diabetic retinopathy (OR = 2.51; 95% CI 1.14–5.53, *P* = 0.022), higher resting SBP ≥ 140 mmHg (OR = 3.14; 95% CI 1.31–8.7.56, *P* = 0.011), and history of palpitations (OR = 2.31; 95% CI 1.08–4.92, *P* = 0.031). Self-report of palpitations (OR = 3.14; 95% CI 1.42–6.95, *P* = 0.005), and higher resting SBP ≥ 140 mmHg (OR = 22.01; 95% CI 1.10–4.42, *P* = 0.043) were associated with OHT.

**Conclusion:**

OH and OHT are common among diabetic individuals in ambulatory diabetes care in southwestern Uganda. Orthostatic BP measurements should be considered as part of routine physical examination to improve detection of OH and OHT, especially among older diabetics with complications of the disease. Future studies to assess the health and prognostic implications of OH and OHT among diabetics in the region are warranted.

## Introduction

Orthostatic hypotension (OH) is a hemodynamic disorder that is characterized by a sustained drop in blood pressure (BP) from supine position to standing [1]. Orthostatic hypertension (OHT), on the other hand is a reverse phenomenon, characterized by an exaggerated sustained rise in BP from supine to standing position [2]. The primary pathophysiological mechanism involved in both OH and OHT is believed to be autonomic nervous system dysfunction [3]. Unlike OHT, OH has been well studied in different populations outside sub-Saharan Africa. The prevalence of OH is age-dependent, and higher in individuals with diabetes mellitus (DM) compared to non-diabetics [4, 5]; prevalence rates ranging from 7 to 64%, have been reported in diabetic populations [6]. Among diabetic individuals, several factors have been found to be associated with OH including age, smoking, duration of diabetes, obesity, hyperlipidemia, hypertension, glycemic control, and coexistence of other microvascular complications of DM [6–8].

OH is recognized as the among most disabling features of autonomic dysfunction [9], and is associated with adverse cardiovascular outcomes, including cardiac failure, stroke, myocardial infarction, and all-cause mortality [10–12]. Patients with OH are prone to syncope, falls, and cognitive impairment due to the resultant cerebral hypo-perfusion [9, 13–15]. Nonetheless, some with abnormal postural blood pressure abnormalities remain asymptomatic because of cerebral blood flow auto-regulatory mechanisms [16]. The concomitant presence of DM and abnormal orthostatic BP responses, has previously been referred to as a “dangerous combination”, because these conditions may portend a much poorer prognosis of cardiovascular complications [6, 17, 18]. Despite being serious and frequent complications, abnormal postural changes in BP are often unrecognized [2, 19].

In addition, some complications such as supine hypertension, usually observed in more than half of diabetics with OH, are challenging to manage and often require expertise of a cardiovascular specialist [20, 21]. Furthermore, there is mounting evidence to suggest that OHT is associated circadian BP abnormalities including labile hypertension during daytime, and extreme dipping at night; the circadian BP abnormalities accelerate hypertensive target organ damage [2].

However, there are very few data on the epidemiology of OH and OHT outside of resource-rich settings. Given the growing evidence of deleterious cardiovascular outcomes for individuals with abnormal orthostatic BP responses and DM [18, 21], it is crucial, that the epidemiology of OH and OHT among those with DM is better understood in more diverse global populations such that complications of these conditions can be considered, to minimize possible cardiovascular risk and improve the quality of life in this patient population [20].

Most earlier studies on abnormal postural changes in BP in diabetic populations have mainly focused on elderly individuals ≥ 70 years, and have taken place outside sub-Saharan Africa (SSA). Data on the burden and correlates of abnormal postural changes in BP in diabetic populations in SSA are limited. We aimed to respond to this gap in the literature by characterizing the prevalence of abnormal orthostatic BP responses, both OH and OHT, and to identify factors associated with these conditions in a relatively younger population of diabetic individuals (18–65 years) in ambulatory DM care in Uganda.

## Methods

### Study population and design

This was a cross-sectional study, conducted at the Diabetes and Endocrinology Clinic of Mbarara Regional Referral Hospital (MRRH) from November 2018 to April 2019. The detailed methods for the parent study that assessed the overall burden of cardiovascular autonomic neuropathy among individuals with diabetes in ambulatory care, and a detailed description of the study population at inclusion, have been published previously [22]. In brief, we included individuals with diabetes aged 18–65 years. We excluded patients with known underlying hepatic, renal, or cardiac diseases. We also excluded patients with known ECG or electrolyte abnormalities, those with acute febrile illnesses, and those actively taking antihypertensive medications such as calcium channel blockers, beta blockers and diuretics in the previous 24 h.

### Study definitions and procedures

We collected clinical and demographic data, using an interviewer administered structured questionnaire. Waist circumference was measured at the level of umbilicus with inelastic tapeline (to the nearest 0.1 cm), at the end of normal expiration. Height and weight of study participants were measured to the nearest 0.1 cm and 0.1 kg respectively. We calculated body mass index as the weight (kg) divided by the square of height (m^2^). Measurements for glycosylated hemoglobin (HbA1c) were performed using an automated high performance liquid chromatography analyzer (Cobas Integra 400, Roche diagnostics, Basel, Switzerland), at Lancet laboratories. Measurements of fasting blood glucose were obtained under aseptic technique, using a Freestyle Glucometer (Abbott Diabetes Care Inc., Maidenhead, UK) after at least 8 h of fasting, by means of capillary blood obtained by finger prick. Diabetes was defined in participants with fasting capillary glucose of ≥ 7.0 mmol/L or those who were already on medication for diabetes mellitus, as per recognized criteria [23]. Diabetic retinopathy was diagnosed using direct ophthalmoscopy and classified as non-proliferative or proliferative diabetic retinopathy, by an ophthalmologist.

We performed resting 12 lead ECG recordings using a portable ECG machine (Edan Instruments, Inc., Hessen, Germany) on study participants, and measured the QT interval from the beginning of the earliest onset of the QRS complex to the end of the T wave, where it crosses the isoelectric line. We used the Bazette’s formula to calculate a heart rate–corrected QT (QTc) [24]. We consider a cut-off value of the QTc interval > 440 ms to be elevated [25].

### Blood pressure measurements and assessment of orthostatic hypotension

All BP recordings were done using an automatic sphygmomanometer in the upper arm (Omron HEM 705 LP, Omron Healthcare, Inc., Bannockburn, IL, USA). The protocol for assessment of OH and OHT included supine BP and standing BP measurements. Supine BP was recorded after 5 min of rest. Standing BP was measured within 3 min of assuming upright standing position. We defined OH in participants with either a ≥ 20 mmHg drop in systolic BP (SBP) or ≥ 10 mmHg drop in diastolic BP (DBP) when assuming an upright position [26]. To define OHT, we considered the cut-off for abnormal systolic orthostatic blood pressure responses of ≥ 20 mmHg, which has been previously proposed for hypertensive orthostatic BP responses [1]. We thus defined systolic OHT in participants with a rise in SBP ≥ 20 mmHg, with or without an accompanying DBP rise; systolic OH was defined in participants with a drop of SBP ≥ 20 mmHg, with or without an accompanying DBP drop. To define diastolic orthostatic hypotensive responses, we considered the cut-off was ≥ 10 mmHg [1] and applied this cut-off for diastolic OHT, since there is no consensus for the numerical threshold for this entity [18]. Thus, we defined diastolic OHT in participants with an increase in diastolic BP ≥ 10 mmHg, with or without an accompanying SBP increase, and diastolic OH in participants with a drop in diastolic BP ≥ 10 mmHg, with or without an accompanying drop in SBP Accordingly, a normal diastolic orthostatic BP response was defined in participants with a change of DBP within the range of − 9 to + 9 mmHg, whereas a normal systolic orthostatic BP response was defined in participants with a change of SBP within the range of − 19 to + 19 mmHg.

Conventional BP measurements were done with participants in sitting positions. We defined hypertension in participants whose BP values were ≥ 140/90 mmHg or who were already on medications for high BP [27]. We calculated pulse pressure as the difference between systolic and diastolic BP measurements. Although there is no widely recognized cut-off for defining high pulse pressure, we adopted the previously suggested threshold of ≥ 65 mm Hg [28].

### Sample size and statistical analyses

For the parent study, which was designed to estimate the prevalence of autonomic neuropathy, we estimated a sample size of 296 participants around a prevalence estimate of cardiovascular autonomic neuropathy (CAN) of 20% [29], with a 5% precision at a 95% level of confidence, after inflation for 10% non-response rate, using Epi Info (version 7.1.4.0, CDC, Atlanta, US). Study data were entered data into EpiData3. (EpiData, Odense, Denmark)**.** We used Stata, version 13 (StataCorp, College Station, Texas, USA) for all statistical analyses.

Our primary outcomes were OH and OHT. First, we determined the prevalence of OH and OHT, as the proportion of participants that met their definitions. Next, we compared differences in clinical and demographic characteristics between participants with OH and OHT and those without. Continuous normally distributed data (presented as mean ± standard deviation) were compared using a two-tailed independent *t*-test; while continuous non-normally distributed data (presented as median ± inter-quartile range) were compared using Wilcoxon rank-sum test. We compared categorical variables using the chi-squared test. Finally, we performed univariate and multivariate logistic regression analyses to identify factors associated with OH and OHT. All variables with a *P*-value less than 0.1, were included in the multivariate models and adjusted for sex, age, BMI, HbA1c, QTc interval, and duration of diabetes, based on biological plausibility. Pulse pressure—derived from SBP and DPB— and history of hypertension, were eliminated from the final multivariate model because of collinearity. We considered statistical significance at a threshold of *P* < 0.05.

## Results

### Characteristics of study participants

The clinical and demographic characteristics of the total cohort have been described previously [22]. In brief, we enrolled a total of 299 participants, with a mean age of 50 years (SD ± 9.8), mean duration of diabetes of 5.8 (SD ± 5.9) years, and mean HbA1c of 9.7% (SD ± 2.6); 70% were female.

Overall, abnormal postural changes in BP were detected in 66 participants (22.1%). Of the 299 participants assessed orthostatic BP responses, 52 (17.4%; 95% CI 13.3–22.2%) met the definition of OH; 43 (14.4%; 95% CI 10.6–18.9%) were classified as having OHT (Table 1).

**Table 1:** Orthostatic blood pressure responses among 299 participants with diabetes aged 18–65 years, southwestern Uganda

Category of orthostatic BP response	Total (N = 299)	
	Frequency (n)	Percent (%)
Normal orthostatic BP response^a^	233	77.9
Abnormal orthostatic BP response^b^	66	22.1
Orthostatic hypotension	52	17.4
Orthostatic hypertension	43	14.4
Diastolic hypertension	38	12.7
Systolic hypotension	23	7.7
Diastolic hypotension	10	3.3
Systolic hypertension	10	3.3

^a^Absence of orthostatic hypertension or orthostatic hypotension

^b^Presence of orthostatic hypertension or orthostatic hypotension

**Fig. 1:**
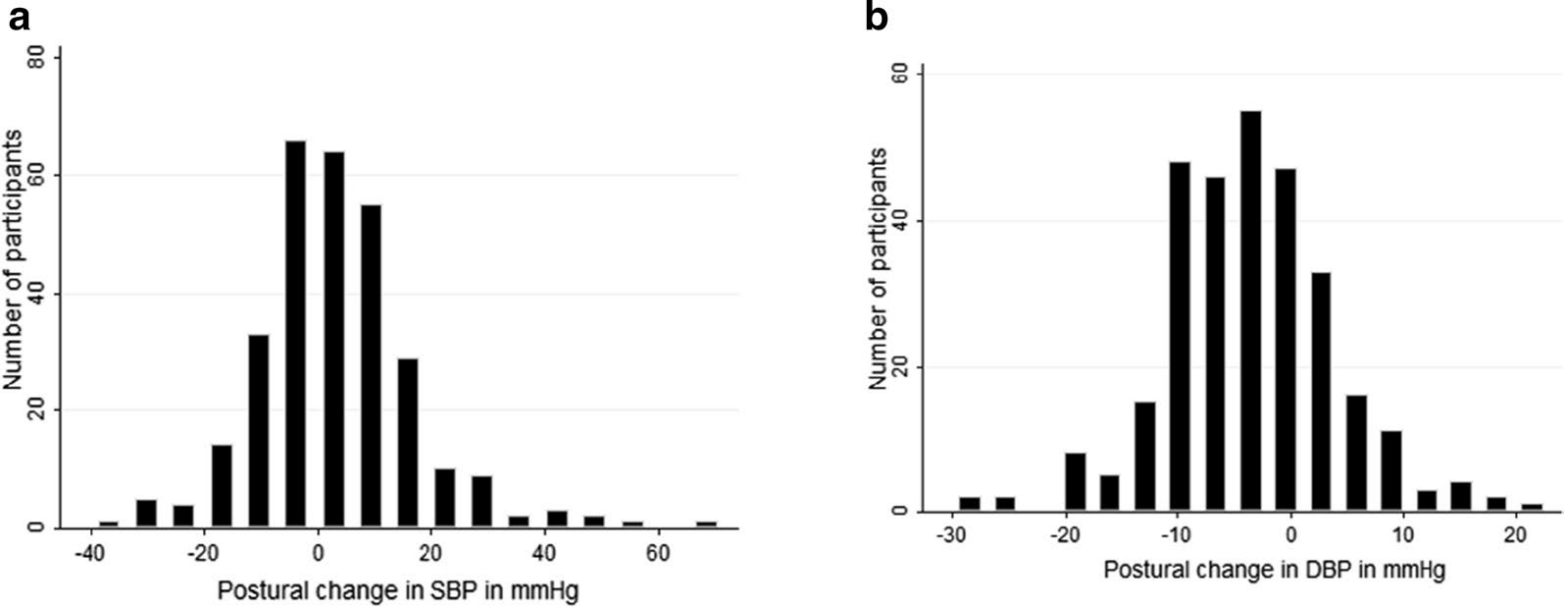
Distribution of systolic (**a**) and diastolic (**b**) orthostatic blood pressure changes among 299 study participants with diabetes aged 18–65 years, southwestern Uganda

Diastolic hypertension was found in 38 participants (12.7%), systolic hypotension in 23 participants (7.7%), while diastolic hypotension and systolic hypertension were found in 10 participants (3.3%) each. The differences between blood pressures from supine to standing followed a normal distribution pattern for both systolic and diastolic BP measurements are shown in Fig. 1. The mean (SD) change for the systolic BP was 3 (± 13) mmHg; for the DBP, the mean change (SD) was -4 (± 8) mmHg.

We found crude differences in the mean age (*P* = 0.002), history of hypertension (*P* = 0.002), history of palpitations (*P* = 0.002), diabetic retinopathy (*P* < 0.001), mean QTc interval (*P* = 0.006) and median duration of diabetes (*P* = 0.031) between those with and without OH. Participants with OH also had significantly higher mean resting systolic BP (*P* < 0.001), higher mean resting diastolic BP (*P* = 0.004) and higher mean pulse pressure (*P* < 0.001) compared with their counterparts with no OH.

The proportion of female participants (*P* = 0.011) and those who reported history of palpitations (*P* < 0.001) was significantly higher in the OHT group than in the group without OHT, as shown in Table 2.

**Table 2:** Characteristics of 299 study participants with diabetes aged 18–65 years, by presence of orthostatic hypotension and orthostatic hypertension, southwestern Uganda

Characteristic	Orthostatic hypotension (OH)		*P* value	Orthostatic hypertension (OHT)		*P* value
	Yes (n = 52)	No (n = 247)		Yes (n = 43)	No (n = 256)	
Age in years, mean (SD)	54 (± 8.3)	49 (± 10)	0.002	54 (± 8.3)	49 (± 10)	0.002
Occupation, n (%)						
Peasant farmer	39 (75.0)	136 (55.1)	0.008	39 (75.0)	136 (55.1)	0.008
Business	7 (13.5)	57 (23.1)	0.124	7 (13.5)	57 (23.1)	0.124
Civil servant	7 (13.5)	44 (17.8)	0.448	7 (13.5)	44 (17.8)	0.448
Female sex, n (%)	38 (73.1)	170 (68.8)	0.545	38 (73.1)	170 (68.8)	0.011
History of smoking, n (%)	11 (21.2)	58 (23.5)	0.717	9 (20.9)	60 (23.4)	0.718
No alcohol use, n (%)	41 (78.9)	168 (68.0)	0.300	30 (69.8)	179 (69.9)	0.975
History of hypertension, n (%)	33 (63.5)	100 (40.5)	0.002	25 (58.1)	108 (42.2)	0.051
Clinical symptoms, n (%)						
Palpitations	36 (69.2)	113 (45.8)	0.002	32 (74.4)	117 (45.7)	< 0.001
Fainting	25 (48.1)	89 (36.0)	0.104	20 (46.5)	94 (36.7)	0.221
Numbness in feet	36 (69.2)	147 (59.5)	0.191	29 (67.4)	154 (60.2)	0.364
Diabetic retinopathy, n (%)			< 0.001			0.103
None	29 (55.8)	202 (81.8)		28 (65.1)	203 (79.3)	
Non-proliferative	16 (30.8)	37 (15)		11 (25.6)	42 (16.4)	
Proliferative	7 (13.5)	8 (3.2)		4 (9.3)	11 (4.3)	
Duration of diabetes in years, median (IQR)	4 (3–12)	4 (1–8)	0.031	4 (1–11)	4 (1–8)	0.553
BMI in kg/m2, mean (SD)	28.1 (± 6.1)	27.3 (± 5.5)	0.350	29.3 (± 6.8)	27.1 (± 5.3)	0.015
Waist circumference in cm, mean (SD)	100 (± 14)	98 (± 13)	0.188	103 (± 3.0)	97 (± 13)	0.019
Fasting blood sugar in mmol/L, mean (SD)	11.0 (± 4.5)	11.2 (± 4.9)	0.750	11.5 (± 4.9)	11.1 (± 4.9)	0.621
HbA1c, mean (SD)	9.6 (± 2.4)	9.7 (± 2.6)	0.671	9.7 (± 2.6)	9.7 (± 2.6)	0.881
QTc interval in ms, mean (SD)	442 (± 21)	431 (± 22)	0.006	439 (± 19)	433 (± 22)	0.049
Resting heart rate in beats/min, mean (SD)	79 (± 15)	76 (± 13)	0.232	78 (± 15)	76 (± 13)	0.507
Pulse pressure in mmHg, mean (SD)	68 (± 22)	52 (± 15)	< 0.001	66 (± 23)	53 (± 16)	< 0.001
Systolic BP in mmHg, mean (SD)	158 (± 25)	138 (± 21)	< 0.001	152 (± 23)	140 (± 22)	0.001
Diastolic BP in mmHg, mean (SD)	91 (± 12)	86 (± 10)	0.004	87 (± 12)	86 (± 10)	0.869

*BP* blood pressure, *BMI* body mass index, *SD* standard deviation, *IQR* interquartile range, *QTc* heart rate corrected QT interval

Participants with OHT had significantly higher mean BMI (*P* = 0.015), higher mean waist circumference (*P* = 0.019), higher mean pulse pressure (*P* < 0.001), higher mean resting systolic BP (*P* = 0.001), and higher mean QTc (*P* = 0.049) than their counterparts (Table 2).

### Correlates of orthostatic hypotension and orthostatic hypertension

In multivariate analysis (Table 3), the factors associated with diagnosis of diabetic OH were older age of 51–65 years (OR = 2.40; 95% CI 1.02–5.67, *P* = 0.046), presence of diabetic retinopathy (OR = 2.51; 95% CI 1.14–5.53, *P* = 0.022), history of palpitations (OR = 2.31; 95% CI 1.08–4.92, *P* = 0.031), and higher resting systolic BP of ≥ 140 mmHg (OR = 3.14; 95% CI 1.31–8.7.56, *P* = 0.011).

**Table 3:** Univariate and multivariate logistic regression analyses for factors associated with orthostatic hypotension, and orthostatic hypertension among 299 participants with diabetes aged 18–65 years, southwestern Uganda

Characteristic	Orthostatic hypotension (OH)			Orthostatic hypertension (OHT)		
	Univariate analysis	Multivariate analysis		Univariate analysis	Multivariate analysis	
	OR (95% CI)	aOR (95% CI)	*P* value	OR (95%CI)	aOR (95%CI)	*P* value
Age category						
18–50 years	Reference	Reference		Reference	Reference	
51–65 years	2.20 (1.16–4.16)	2.40 (1.02–5.67)	0.046	1.37 (0.71–2.64)	1.26 (0.50–3.16)	0.630
Sex						
Male	Reference	Reference		Reference	Reference	
Female	1.23 (0.63–2.40)	0.76 (0.32–1.82)	0.535	3.07 (1.25–7.55)	2.67 (0.96–7.55)	0.061
History of hypertension^a^						
No	Reference			Reference		
Yes	2.55 (1.37–4.74)			1.90 (0.99–3.66)		
History of palpitations						
No	Reference	Reference		Reference	Reference	
Yes	2.67 (1.41–5.06)	2.31 (1.08–4.92)	0.031	3.46 (1.67–7.16)	3.14 (1.42–6.95)	0.005
Body mass index						
< 25 kg/m^2^	Reference	Reference		Reference	Reference	
≥ 25 kg/m2	1.14 (0.13–9.84)	1.29 (0.12–13.87)	0.832	1.18 (0.59–2.35)	1.04 (0.42–2.53)	0.937
Diabetic retinopathy						
No	Reference	Reference		Reference	Reference	
Yes	3.56 (1.89–6.72)	2.51 (1.14–5.53)	0.022	1.71 (1.03–2.85)	1.24 (0.56–2.75)	0.598
Duration of diabetes						
< 5 years	Reference	Reference		Reference	Reference	
5–10 years	0.62 (0.27–1.44)	0.49 (0.19–1.27)	0.143	0.86 (0.38–1.96)	0.64 (0.26–1.58)	0.331
> 10 years	2.50 (1.23–5.08)	1.21 (0.48–3.06)	0.689	1.67 (0.96–3.71)	1.06 (0.40–2.80)	0.903
Pulse pressure^a^						
< 65 mmHg	Reference			Reference		
≥ 65 mmHg	4.37 (2.32–8.21)			2.69 (1.37–5.30)		
Resting systolic BP						
< 140 mmHg	Reference	Reference		Reference	Reference	
≥ 140 mmHg	5.23 (2.51–10.90)	3.14 (1.31–8.7.56)	0.011	2.53 (1.26–5.08)	2.01 (1.10–4.42)	0.043
Resting diastolic BP						
< 90 mmHg	Reference	Reference		Reference	Reference	
≥ 90 mmHg	2.42 (1.32–4.45)	1.60 (0.75–3.43)	0.224	1.37 (0.72–2.64)	0.88 (0.40–1.97)	0.764
QTc interval						
< 440 ms	Reference	Reference		Reference	Reference	
≥ 440 ms	2.9 (1.58–5.39)	2.95 (0.99–6.14)	0.052	1.87 (0.97–3.58)	1.31 (0.63–2.69)	0.471
Glycosylated haemoglobin (HbA1c)						
≤ 7.5%	Reference	Reference		Reference	Reference	
> 7.5%	0.98 (0.87–1.10)	1.06 (0.45–2.45)	0.901	1.01 (0.89–1.14)	0.75 (0.31–1.75)	0.499

*aOR* adjusted odds ratio, *OR* odds ratio, *OH* orthostatic hypotension, *OHT* orthostatic hypertension, *BP* blood pressure; *QTc* heart rate corrected QT interval, *CI* Confidence interval

^a^Eliminated from the multivariate model because of collinearity

Having history of palpitations (OR = 3.14; 95% CI 1.42–6.95, *P* = 0.005) and higher resting SBP ≥ 140 mmHg (OR = 2.01; 95% CI 1.10–4.42, *P* = 0.043) were associated with presence of OHT in the multivariate analysis (Table 3).

## Discussion

We detected a high prevalence of abnormal postural changes in BP among persons in ambulatory care for DM in Uganda: approximately 17% had OH and 14% had OHT. Notably, risk factors for falls and other comorbidities, including older age, high SBP, palpitations, and retinopathy were significantly associated with OH; high SBP and history of palpitations were each significantly associated with OHT. On balance, our data suggest a need to systematically and routinely detect these abnormal orthostatic responses in BP, so as to improve quality of life in this patient population, particularly in older diabetics with other complications.

With no published data currently available for comparison from sub-Saharan Africa, the prevalence of abnormal postural changes in BP in this study population is similar to previously reported findings from studies among individuals with type 1 diabetes and longstanding disease in USA [30] and older-aged type 2 diabetic individuals in Sweden, Brazil and Japan [18, 31, 32]. A population-based study among adult persons in Japan reported a similar prevalence of OH of 16%; in the same study, however, the prevalence of OHT was very low (1.1%) [33]. Nonetheless, even higher prevalence rates of OH than ours have been reported in elderly individuals with diabetes in France and China [34, 35]. However, it is challenging to compare prevalence rates of OH and OHT because of the different definitions for these clinical entities used across different studies. Some earlier studies used SBP cut-offs of 10 mmHg [31], or a change of DBP from < 90 to ≥ 90 mmHg or an increase of SBP from < 140 to ≥ 140 mmHg after standing from supine position [30, 32]. Nevertheless, our findings highlight that abnormal orthostatic BP reactions (both OH and OHT) are common and unrecognized complications among individuals with diabetes in Uganda.

We found that individuals with higher resting SBP (≥ 140 mmHg) had higher odds of OH, and OHT. This is in agreement with previous studies that have reported association between resting SBP and abnormal orthostatic BP responses [33, 34, 36–40]. Although the pathophysiological mechanisms involved in OHT and OH are largely unclear, both are believed to share similar pathophysiological pathways with autonomic nervous system dysfunction recognized as the primary pathophysiological disturbance in both disorders [3]. In individuals with diabetes and hypertension, the risk for developing OHT may be amplified because both diabetes and hypertension enhance activation of the autonomic nervous system [3]. Moreover, it has been hypothesized that OHT may be a form of masked hypertension or prehypertension among non-hypertensive individuals [40]. Thus, normotensive individuals with diabetes with OHT should be followed up for possible development of hypertension.

Although the precise mechanism of OH is not well studied in this population, we postulate that OH may be a manifestation of autonomic neuropathy, given the high prevalence of cardiovascular autonomic dysfunction detected in this same population [22]. This hypothesis is further supported by the association between increasing age and other microvascular complications (e.g., retinopathy) that are known to be more frequent in neurogenic type of OH [33, 38], probably due to age-related autonomic dysfunction and metabolic insults resulting from chronic hyperglycemia.

Current guidelines for care and management of diabetes in Uganda and other developing countries in sub-Saharan Africa do not routinely recommend screening for orthostatic BP abnormalities among individuals with diabetes in ambulatory care. Because routine measurement of BP is a non-invasive and low-cost procedure to identify individuals with abnormal orthostatic BP changes, our data suggest that routine comprehensive physical examinations in diabetic individuals could serve as a scalable mechanism to improve detection of OH and OHT. Future work should focus on how detection of OH and OHT with subsequent optimization of hemodynamics may improve quality of life and reduce the risk for excess cardiovascular morbidity and mortality associated with these complications.

### Limitations

The findings from this study should be interpreted in consideration of our study limitations. First, our study population lacked individuals older than 65 years, based on the exclusion criteria of the parent study which assessed the overall burden of cardiovascular autonomic neuropathy among individuals with diabetes in ambulatory care. This may have led to underestimation of the prevalence of the OH and OHT, and biased our associations towards the null. Generalizability should be restricted similarly to those with diabetes and in ambulatory care in similar peri-urban African settings. Second, the cross-sectional study design limited us from assigning time causal directionally between our exposures and outcomes. Third, we did not comprehensively assess complications of OH and OHT including orthostatic intolerance and associated falls. Thus, we cannot evaluate the clinical implications of these abnormal orthostatic BP responses in the study population. Further studies are required to assess the prognostic and clinical implications of these orthostatic abnormalities in this patient population in the region. Despite these limitations, our study is among the first studies to provide useful epidemiological data on OH and OHT among diabetic individuals in sub-Saharan African region.

## Conclusions

OH and OHT are common among individuals with diabetes in ambulatory care in southwestern Uganda. Higher SBP, history of palpitations, coexistence of diabetic retinopathy, and older age were key correlates of the orthostatic BP abnormalities. Future work should elucidate how these conditions impact the health and quality of life of those with DM in this region. Given the ease and low cost of performing orthostatic BP measurements in clinical settings, we recommend that clinical guidelines for care of persons with diabetes consider including screening for orthostatic abnormalities in BP into routine physical examinations to improve detection and management of OH and OHT in the region, especially among diabetics with hypertension, older age, and coexisting microvascular complications. Timely management of OH and OHT may potentially minimize future adverse cardiovascular events in this patient population, given that co-existing DM and orthostatic abnormalities in BP portent a much poorer prognosis.

## Data Availability

The datasets generated and analyzed during the study are available from the corresponding author on request.

## Abbreviations

aOR: Adjusted odds ratio
BMI: Body mass index
BP: Blood pressure
DBP: Diastolic blood pressure
CAN: Cardiovascular autonomic neuropathy
CVD: Cardiovascular disease
CI: Confidence interval
ECG: Electrocardiogram
HbA1c: Glycosylated hemoglobin
IQR: Inter-quartile range, Mbarara Regional Referral Hospital
OH: Orthostatic hypotension
OHT: Orthostatic hypertension
OR: Odds ratio
QTc: Heart rate corrected QT interval
SBP: Systolic blood pressure
SD: Standard deviation
